# Supplementary material for: The contribution of corporate initiatives to global renewable electricity deployment
Source: Nat Commun. 2023 Aug 4;14:4678. doi: 10.1038/s41467-023-40356-0 (PMC10403614; doi:10.1038/s41467-023-40356-0)
Supplement: Supplementary file 1 — Supplementary Information [file 41467_2023_40356_MOESM1_ESM.pdf]

Supplementary Information for

The contribution of corporate initiatives to global renewable electricity deployment

Florian Egli[1,2], Rui Zhang[1], Victor Hopo[1], Tobias Schmidt[1,3], Bjarne Steffen[3,4]

[1] Energy and Technology Policy Group, ETH Zurich, Switzerland
[2] Institute for Innovation and Public Purpose, UCL, UK
[3] Institute for Science, Technology and Policy, ETH Zurich, Switzerland
[4] Climate Finance Policy Group, ETH Zurich, Switzerland

Figures

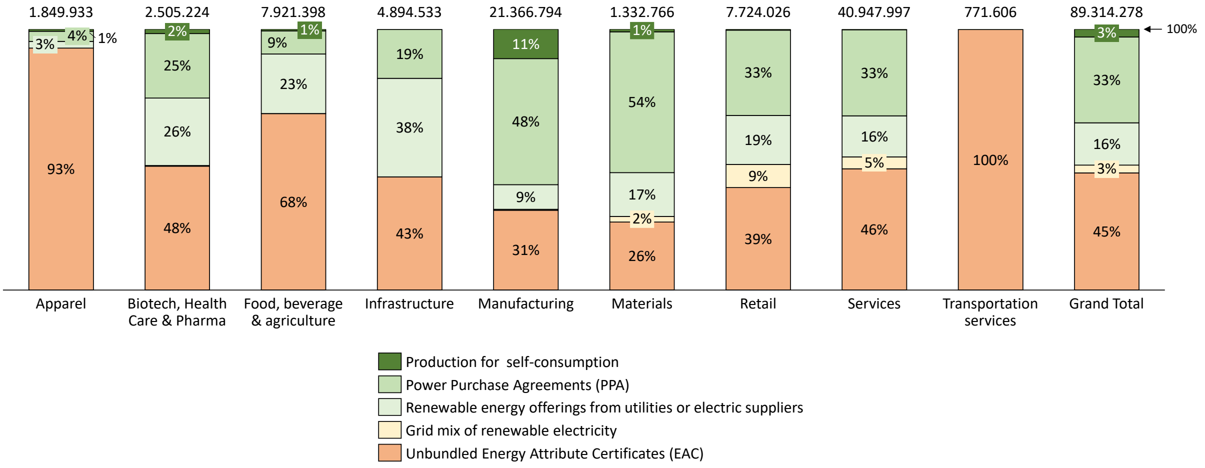

Supplementary Figure 1: Sourcing strategies by sectors

Tables

Supplementary Table 1: RE100 demand shares of global total in 2018.

	**Total generation**	
**RE100**	*Renewable (6,500 TWh)*	*Total (24,800 TWh)*
Renewable (92 TWh)	1.4%	0.4%
Total (227 TWh)	3.5%	0.9%

Supplementary Table 2: RE100 induced electricity demand in percent of total RE generation. Shown for countries where the share exceeds 10%.

**Country**	**Electricity demand of RE100 companies in percent of total domestic RE generation**
Hong Kong SAR, China	476.2%
Puerto Rico	169.6%
Saudi Arabia	121.4%
Bahrain	80.9%
Singapore	62.8%
Trinidad and Tobago	45.4%
Oman	43.7%
Botswana	41.3%
Hungary	20.8%
Netherlands	19.7%
United Arab Emirates	16.4%
Qatar	16.4%
Kuwait	16.4%
Israel	14.8%
United Kingdom	14.6%
Japan	13.6%
United States	11.8%
South Africa	11.4%
Ireland	11.3%
Mexico	10.7%

Supplementary Table 3: Sourcing strategy descriptions. Source: IRENA 2018, p. 15-16[30].

Sourcing strategy	Description
**Corporate power purchase agreement (PPA)**	A company enters into a contract with an independent power producer, a utility or a financier and commits to purchasing a specific amount of renewable electricity, or the output from a specific asset, at an agreed price and for an agreed period of time.
**Unbundled energy attribute certificate (EAC)**	A company purchases attribute certificates of renewable energy separately "unbundled" from its electricity. Examples of certificate systems are guarantees of origin (GOs) and renewable energy certificates (RECs).
**Renewable energy offerings from utilities or electric suppliers**	A company purchases renewable electricity from its utility either through green premium products or through a tailored renewable electricity contract, such as a green tariff programme.
**Production for self-consumption**	A company invests in its own renewable energy systems, on-site or off-site, to produce electricity primarily for self-consumption.
**Grid mix of renewable electricity**	A passive approach in which consumption is based on the average renewable electricity content available in the grids from which companies source their electricity.

Supplementary Table 4: Sectoral classification according to CDP's Activity Classification System.

Sector	Description
**Apparel**	Apparel design, textiles & fabric goods
**Biotech, health care & pharma**	Biotech & pharma, health care provision, medical equipment & supplies
**Food, beverage & agriculture**	Crop farming, fish & animal farming, food & beverage processing, logging & rubber tapping, tobacco
**Hospitality**	Bars, hotels & restaurants, entertainment facilities
**Infrastructure**	Constructure, energy utility networks, land & property ownership & development, non-energy utilities
**Manufacturing**	Electrical & electronic equipment, leisure & home manufacturing, light manufacturing, metal products manufacturing, paper products & packaging, plastic product manufacturing, powered machinery, renewable energy equipment, transportation equipment, wood & rubber product
**Materials**	Cement & concrete, chemicals, metal smelting, refining & forming, metallic mineral mining, other materials, other mineral mining, wood & paper materials
**Retail**	Convenience retail, discretionary retail, trading, wholesale, distribution, rental & leasing
**Services**	Commercial & consumer services, financial services, industrial support services, IT & software development, media, telecommunications & data services, other services, print & publishing services, specialized professional services, web & marketing services
**Transportation services**	Air transport, intermodal transport & logistics, marine transport, rail transport, road transport
