# Supplementary material for: The contribution of corporate initiatives to global renewable electricity deployment
Source: Nat Commun. 2023 Aug 4;14:4678. doi: 10.1038/s41467-023-40356-0 (PMC10403614; doi:10.1038/s41467-023-40356-0)
Supplement: Supplementary file 3 — Reporting Summary [file 41467_2023_40356_MOESM3_ESM.pdf]

nature portfolio

Corresponding author(s): Florian Egli

Last updated by author(s): Jun 12, 2023

Reporting Summary

Nature Portfolio wishes to improve the reproducibility of the work that we publish. This form provides structure for consistency and transparency in reporting. For further information on Nature Portfolio policies, see our Editorial Policies and the Editorial Policy Checklist.

Statistics

For all statistical analyses, confirm that the following items are present in the figure legend, table legend, main text, or Methods section.

n/a	Confirmed	
☐	☒	The exact sample size (*n*) for each experimental group/condition, given as a discrete number and unit of measurement
☒	☐	A statement on whether measurements were taken from distinct samples or whether the same sample was measured repeatedly
☒	☐	The statistical test(s) used AND whether they are one- or two-sided<br>*Only common tests should be described solely by name; describe more complex techniques in the Methods section.*
☒	☐	A description of all covariates tested
☒	☐	A description of any assumptions or corrections, such as tests of normality and adjustment for multiple comparisons
☒	☐	A full description of the statistical parameters including central tendency (e.g. means) or other basic estimates (e.g. regression coefficient) AND variation (e.g. standard deviation) or associated estimates of uncertainty (e.g. confidence intervals)
☒	☐	For null hypothesis testing, the test statistic (e.g. F, t, r) with confidence intervals, effect sizes, degrees of freedom and P value noted<br>*Give P values as exact values whenever suitable.*
☒	☐	For Bayesian analysis, information on the choice of priors and Markov chain Monte Carlo settings
☒	☐	For hierarchical and complex designs, identification of the appropriate level for tests and full reporting of outcomes
☒	☐	Estimates of effect sizes (e.g. Cohen's *d*, Pearson's *r*), indicating how they were calculated

Our web collection on statistics for biologists contains articles on many of the points above.

Software and code

Policy information about availability of computer code

Data collection	No code was used to collect the data.
Data analysis	No statistical software was used. Data processing, harmonization and analysis took place in Microsoft Excel 365, version 2208.

For manuscripts utilizing custom algorithms or software that are central to the research but not yet described in published literature, software must be made available to editors and reviewers. We strongly encourage code deposition in a community repository (e.g. GitHub). See the Nature Portfolio guidelines for submitting code & software for further information.

Data

Policy information about availability of data

All manuscripts must include a data availability statement. This statement should provide the following information, where applicable:

- Accession codes, unique identifiers, or web links for publicly available datasets
- A description of any restrictions on data availability
- For clinical datasets or third party data, please ensure that the statement adheres to our policy

Data to reproduce the figures is available on Figshare. The underlying company–country electricity demand matrix (allocation of electricity demand from company to countries) and other more granular data is partly based on data from CDP (combined with data from other sources), and reproduction of the CDP data by any third party is forbidden by CDP license terms. The data from CDP will be made available by the corresponding authors upon reasonable request given the requesting party has the permission from CDP.

Field-specific reporting

Please select the one below that is the best fit for your research. If you are not sure, read the appropriate sections before making your selection.

☐ Life sciences ☒ Behavioural & social sciences ☐ Ecological, evolutionary & environmental sciences

For a reference copy of the document with all sections, see nature.com/documents/nr-reporting-summary-flat.pdf

Behavioural & social sciences study design

All studies must disclose on these points even when the disclosure is negative.

Study description	The study compiled a company-level dataset on (renewable) electricity demand by country. The dataset draws on a variety of data sources (see below). Based on this dataset we conducted a series of descriptive quantitative analyses to quantify the impact of RE100 on the global energy transition. We did not employ statistical methods.
Research sample	Our "population" of interest is the entirety of the 211 RE100 member companies as per the end of 2019. We were able to find detailed electricity data on 185 of these companies, which constitute our sample. We consider this sample representative of RE100 given the data availability.
Sampling strategy	Our sample is constrained by data availability. Within data availability, we strived to achieve maximum coverage of all RE100 member companies. We therefore did not employ a sampling procedure but aimed to leverage all available data sources to get as closely as possible to the full "population" of interest, i.e., all RE100 member companies.
Data collection	We used a variety of public and proprietary data sources to compile the dataset for the analysis. We used data from CDP (https://www.cdp.net/en), namely the climate change questionnaire, which can be downloaded from the website. We further used proprietary company-level data from ORBIS (https://www.bvdinfo.com/en-gb/) as referenced in the methods. Finally, we used publicly available data sources, such as company reports, UN population data and data from the World Bank and the UN on GDP. All data was collected and extracted manually according to the 4-eye principle by two co-authors aware of the research questions to compile the dataset for the analysis in Microsoft Excel.
Timing	Data collection took place between April 2020 and December 2021 with the majority of the effort being conducted between April 2020 and September 2020.
Data exclusions	No data was excluded in the collection. We excluded 23 countries/jurisdictoins with a population below 1 million from the analysis to improve the readability and interpretability of the results as described in the methods.
Non-participation	No participants involved.
Randomization	No experimental procedure or statistical inference conducted. The study collected data on the entire "population" of interest (see research sample). Therefore, randomization is not applicable.

Reporting for specific materials, systems and methods

We require information from authors about some types of materials, experimental systems and methods used in many studies. Here, indicate whether each material, system or method listed is relevant to your study. If you are not sure if a list item applies to your research, read the appropriate section before selecting a response.

Materials & experimental systems

n/a	Involved in the study	
☒	☐	Antibodies
☒	☐	Eukaryotic cell lines
☒	☐	Palaeontology and archaeology
☒	☐	Animals and other organisms
☒	☐	Human research participants
☒	☐	Clinical data
☒	☐	Dual use research of concern

Methods

n/a	Involved in the study	
☒	☐	ChIP-seq
☒	☐	Flow cytometry
☒	☐	MRI-based neuroimaging
